# Announcements

**Published:** 2015-05-01

## Lyme Disease Awareness Month — May 2015

Lyme disease is a multisystem disease caused by the spirochete *Borrelia burgdorferi*. The organism is transmitted through the bite of certain species of blacklegged ticks (*Ixodes* spp.). In 2013, state and local health departments reported approximately 35,000 cases of Lyme disease to CDC, making it the fifth most commonly reported nationally notifiable condition (1). Research suggests that as many as 300,000 persons in the United States might be diagnosed and treated for Lyme disease each year (2). As with other vectorborne diseases, the geographic distribution of Lyme disease is highly regional. Approximately 95% of confirmed Lyme disease cases are reported from 14 states in the upper Midwest, New England, and the mid-Atlantic states (3). Infection is most common among children aged 5–15 years and adults aged 40–60 years (4).

To assist health care providers in diagnosing and treating Lyme disease and other tickborne diseases, CDC has released the booklet Tickborne Diseases of the United States: A Reference Guide for Health Care Providers (5) and a corresponding cellular telephone and tablet application (6). In addition, free continuing education credits are available (information available at http://emergency.cdc.gov/coca/calls/2014/callinfo_041014.asp).

Residents and travelers in areas where Lyme disease is common should take preventive measures, especially during May–July when the risk is greatest. To help prevent Lyme disease, CDC recommends avoiding areas with tall grass and brush where ticks are common; applying repellents that contain at least 20%–30% N,N-diethyl-m-toluamide (DEET); wearing clothing treated with 0.5% permethrin; showering soon after coming indoors; and seeking health care promptly if symptoms of Lyme disease develop, including fever, rash, and muscle or joint pain (7).
